# Pilot implementation of newborn hearing screening programme at four hospitals in southern Thailand

**DOI:** 10.2471/BLT.18.220939

**Published:** 2019-09-03

**Authors:** Pittayapon Pitathawatchai, Wandee Khaimook, Virat Kirtsreesakul

**Affiliations:** a Department of Otolaryngology, Faculty of Medicine, Prince of Songkla University, 15 Karnjanavanich road, Hat Yai, Chang Wat Songkhla, 90110, Thailand.

## Abstract

**Objective:**

To determine the effectiveness and benefit of a universal newborn hearing screening programme at four different hospitals in southern Thailand, between January and July 2017.

**Methods:**

One screener per hospital recorded demographic data of all newborns and their exposure to risk of hearing loss, and evaluated their hearing by transient otoacoustic emission technology. Those who demonstrated bilateral moderate to profound hearing loss at both a first and second screening were referred for diagnostic assessment. Those with confirmed hearing loss received treatment and regular follow-up appointments, and their speech development was assessed at 1 year of age. We determined effectiveness by comparing our achieved coverage and proportion of follow-up and referrals with benchmarks set by the American Academy of Pediatrics (≥ 95%, ≥ 95% and ≤ 4%, respectively), and determined benefit by calculating the composite language scores of hearing-impaired infants who received early intervention.

**Findings:**

We screened 6140 eligible newborns, and achieved a screening coverage of 95.4% (5859/6140), lost 25.7% (63/245) and 22.0% (9/41) to follow-up at the second screening and diagnostic assessment stages, respectively, and obtained an overall proportion of referrals of 0.7% (41/6140). Twelve infants were confirmed as having hearing loss and received early intervention; nine (75%) demonstrated normal speech development by their first birthday. Our universal hearing screening yielded a prevalence of sensorineural hearing loss of less than 0.1% (3/6140).

**Conclusion:**

Although ineffective by American Academy of Pediatrics standards, we demonstrated the benefit of early intervention in infants diagnosed with hearing loss.

## Résumé

**Objectif:**

Déterminer l'efficacité et l'utilité d'un programme de dépistage auditif universel chez les nouveau-nés mis en œuvre dans quatre hôpitaux du sud de la Thaïlande entre janvier et juillet 2017.

**Méthodes:**

Dans chaque hôpital, un examinateur a pris note des données démographiques de tous les nouveau-nés et de leur exposition au risque de perte auditive, et a évalué leur audition à l'aide d'un appareil à otoémissions acoustiques transitoires. Ceux qui présentaient une perte auditive bilatérale modérée à profonde lors d'un premier dépistage, puis d'un second, ont fait l'objet d'une évaluation diagnostique. Ceux pour lesquels la perte auditive a été confirmée ont reçu un traitement et des rendez-vous de suivi réguliers, et leur développement langagier a été évalué à leur 1er anniversaire. Nous avons déterminé l'efficacité en comparant la couverture obtenue et la proportion de suivis et d'aiguillages aux données de référence établies par l'Académie américaine de pédiatrie (respectivement ≥ 95%, ≥ 95% et ≤ 4%), et déterminé l'utilité en calculant les résultats langagiers combinés des nourrissons déficients auditifs qui avaient reçu une intervention précoce.

**Résultats:**

Nous avons sélectionné 6140 nouveau-nés admissibles, et atteint une couverture de dépistage de 95,4% (5859/6140), perdu de vue 25,7% (63/245) et 22,0% (9/41) des nouveau-nés lors du second dépistage et de l'évaluation diagnostique, respectivement, et obtenu une proportion générale d'aiguillages de 0,7% (41/6140). La perte auditive a été confirmée chez douze nourrissons qui ont reçu une intervention précoce; neuf (75%) présentaient un développement langagier normal à leur 1er anniversaire. Notre dépistage auditif universel a permis d'obtenir une prévalence de la perte auditive neurosensorielle inférieure à 0,1% (3/6140).

**Conclusion:**

Bien que jugée inefficace par l'Académie américaine de pédiatrie, nous avons démontré l'utilité d'une intervention précoce chez les nourrissons diagnostiqués avec une perte auditive.****

## Resumen

**Objetivo:**

Determinar la efectividad y el beneficio de un programa universal de exámenes de audición para recién nacidos en cuatro hospitales diferentes del sur de Tailandia, entre enero y julio de 2017.

**Métodos:**

Un examinador por hospital registró los datos demográficos de todos los recién nacidos y su exposición al riesgo de pérdida de audición, y evaluó su audición mediante la tecnología de emisión otoacústica de evocación transitoria. Aquellos que demostraron una pérdida auditiva bilateral de moderada a profunda tanto en el primer como en el segundo examen fueron remitidos para una evaluación diagnóstica. Aquellos con pérdida auditiva confirmada recibieron tratamiento y citas regulares de seguimiento, y su desarrollo del habla fue evaluado a 1 año de edad. Se determinó la efectividad comparando la cobertura lograda y la proporción de seguimiento y derivaciones con los puntos de referencia establecidos por la Academia Estadounidense de Pediatría (≥ 95 %, ≥ 95 % and ≤ 4 %, respectivamente), y se determinó el beneficio mediante el cálculo de puntajes de lenguaje compuesto de los infantes con deficiencias auditivas que recibieron la intervención temprana.

**Resultados:**

Examinamos a 6 140 recién nacidos elegibles y logramos una cobertura de 95,4 % (5 859/6 140), perdimos 25,7 % (63/245) y 22,0 % (9/41) para el seguimiento en las segundas etapas de exámenes de detección y de evaluación diagnóstica, respectivamente, y obtuvimos una proporción general de remisiones de 0,7 % (41/6 140). Se confirmó que doce infantes tenían pérdida auditiva y recibieron intervención temprana; nueve (75 %) demostraron un desarrollo normal del habla para su primer cumpleaños. Nuestro examen de audición universal arrojó una prevalencia de pérdida auditiva neurosensorial de menos del 0,1 % (3/6 140).

**Conclusión:**

Aunque los estándares de la Academia Americana de Pediatría no son efectivos, demostramos el beneficio de la intervención temprana en infantes diagnosticados con pérdida auditiva.

## ملخص

**الغرض:**

تحديد فعالية برنامج فحص السمع الشامل لدى حديثي الولادة وفائدته في أربعة مستشفيات مختلفة في جنوب تايلند، في الفترة ما بين شهر يناير/كانون ثاني إلى شهر يوليو/تموز 2017.

**الطريقة:**

سجل فاحص واحد لكل مستشفى قام بتسجيل البيانات الديموغرافية لجميع حديثي الولادة وتعرضهم لخطر فقدان السمع، وتقييم سمعهم بواسطة تقنية الانبعاثات الصوتية العابرة. وتم إحالة أولئك الذين أظهروا ضعف سمع ثنائي معتدل إلى شديد في كل من الفحص الأول والثاني للتقييم التشخيصي. وتلقى هؤلاء الذين عانوا من فقدان مؤكد للسمع العلاج بالإضافة إلى تحديد مواعيد متابعة منتظمة، وتم تقييم تطور الكلام لديهم عند بلوغ عام واحد. لقد حددنا الفعالية عن طريق مقارنة التغطية المحرزة ونسبة المتابعة والإحالات، وفقاً للمعايير التي وضعتها الأكاديمية الأمريكية لطب الأطفال (≤ 95%، و≤ 95%، و≥ 4%، على التوالي)، كما حددنا الفوائد عن طريق حساب درجات اللغة المركبة للرضع ضعاف السمع الذين تلقوا التدخل المبكر.

**النتائج:**

قمنا بفحص 6140 طفل حديث ولادة مؤهل، وحققنا تغطية فحص بنسبة 95.4% (5859/6140)، وفقدنا 25.7% (63/245)، ومتابعة نسبة 22.0% (9/41) في مراحل الفحص الثاني والتقييم التشخيصي، على التوالي، وحصلنا على نسبة إجمالية للإحالات بلغت 0.7% (41/6140). تم تأكيد إصابة إثني عشر رضيعًا بفقدان السمع وتلقيهم للتدخل المبكر؛ أظهر تسعة منهم (75%) تطور طبيعي في الكلام بحلول عيد ميلادهم الأول. وأسفر فحص السمع الشامل لدينا عن انتشار فقدان السمع الحسي العصبي بنسبة أقل من 0.1% (3/6140).

**الاستنتاج:**

على الرغم من عدم فعالية معايير الأكاديمية الأمريكية لطب الأطفال، إلا أننا قد أظهرنا فائدة التدخل المبكر عند الرضع الذين تم تشخيص إصابتهم بفقدان السمع.

## 摘要

**目的:**

确定 2017 年 1 月至 7 月泰国南部地区四所不同类型医院新生儿听力筛查方案的成效和效益。

**方法:**

每家医院安排一名筛查员记录所有新生儿的人口统计数据及其失聪风险，并采用瞬态耳声传射技术评估他们的听力水平。在第一次和第二次筛查中均显示双耳中度至重度失聪的患者需要转诊以进行诊断评估。已确诊的失聪患者需接受治疗并定期随访，且需在 1 岁时对其言语发育进行评估。我们通过将随访和转诊患者的已达成覆盖率和比例与美国儿科学会设定的基准（分别为≥95%、≥95% 和≤ 4%）进行对比来确定疗效，并通过计算接受早期干预的失聪婴儿的综合语言评分来确定效益。

**结果:**

我们筛查了 6140 名符合条件的新生儿，实现了 95.4% (5859/6140) 的筛查覆盖率，在第二次筛查和诊断评估阶段的失访率分别为 25.7% (63/245) 和 22.0% (9/41)，转诊总比例为 0.7% (41/6140)。12 名婴儿被确诊为失聪，并接受了早期干预；其中，9 名婴儿 (75%) 在一岁生日前的语言发育表现为正常。普遍听力筛查的成效在于感音神经性听力损失的发病率低于 0.1% (3/6140)。

**结论:**

尽管按照美国儿科学会的标准，这并无效果，但我们证明了早期干预对被诊断为患有听力损失的婴儿有所益处。****

## Резюме

**Цель:**

Определение эффективности и пользы программы всеобщего скринингового обследования слуха новорожденных, проводившейся в четырех разных больницах южного Таиланда в период с января по июль 2017 года.

**Методы:**

Специалист по скринингу регистрировал в каждой из больниц демографические данные всех новорожденных и их подверженность риску потери слуха, а также оценивал их слух методом транзиторной отоакустической эмиссии. Младенцы, у которых отмечалась двусторонняя потеря слуха от умеренной до глубокой степени как при первом, так и при втором скрининге, направлялись на диагностическое обследование. Детям с подтвержденным диагнозом потери слуха назначалось лечение с регулярным наблюдением у врача, и по достижении ими возраста 1 года проводилась оценка речевого развития. Авторы определяли эффективность путем сравнения степени достигнутого охвата и доли направлений к специалистам и на контрольные визиты с показателями, установленными Американской академией педиатрии (≥95, ≥95 и ≤4% соответственно), а пользу определяли посредством расчета композитной оценки речевого развития детей с нарушениями слуха, получивших помощь в результате раннего вмешательства.

**Результаты:**

Авторы провели скрининговое обследование 6140 новорожденных, соответствующих критериям для оценки, и получили показатели, составляющие 95,4% (5859 из 6140 детей) для степени охвата, 25,7% (63 из 245 детей) для потерь и 22,0% (9 из 41 ребенка) для явки на контрольный визит при втором скрининге и диагностическом обследовании, и, соответственно, достигли доли направления к специалистам, составившей 0,7% (41 из 6140 детей). У двенадцати младенцев диагноз потери слуха был подтвержден и было проведено ранее вмешательство, и девять из них (75%) демонстрировали нормальное речевое развитие к возрасту 1 года. Согласно данным универсального скринингового обследования слуха, распространенность нейросенсорной потери слуха составила менее 0,1% от всех случаев (3 из 6140 детей).

**Вывод:**

Несмотря на отсутствие эффективности исследования в соответствии со стандартами Американской академии педиатрии, авторы смогли продемонстрировать пользу раннего вмешательства для детей, у которых была диагностирована потеря слуха.

## Introduction

Congenital hearing loss affects 1–3 neonates per 1000 live births,^1^ and 2–4% of newborns who are exposed to potential risk factors in a neonatal intensive care unit can develop sensorineural hearing loss.^1^^,^^2^ Hearing loss can adversely affect the speech and language development of children if not detected at an early stage and treated.^3^ To limit these adverse effects related to hearing loss, the Joint Committee on Infant Hearing^4^ recommend universal newborn hearing screening.

Hearing screening programmes for newborns have been implemented in many countries. The provision of such programmes is dependent on resources, legislative support and the availability of qualified personnel and competent audiological services. In the United States of America, screening coverage in 2016 was approximately 98% of all live births.^5^ In less wealthy nations, such as the Philippines, a national hearing screening programme for newborns began in 2009 after relevant legislation was passed.^6^ More than 60 districts in India have established hearing screening programmes for newborns since 2006.^7^ Cultural variations must be considered when planning such a programme. For example, in Nigeria most births occur outside hospitals. A community-based hearing screening programme incorporated within the regular immunization schedule was therefore found to be feasible and effective.^8^

A universal newborn hearing screening programme is not included in the public health service in Thailand. Although the Royal College of Otolaryngologists-Head and Neck Surgeons of Thailand and the Ministry of Public Health discussed and promoted such a programme in 2017, no commitment on the date of its launch was provided due to a lack of resources. The number of qualified audiology providers in Thailand is severely limited. Only 226 audiologists and 98 speech-language pathologists^9^ were available in 2017 to serve a population of over 66 million with 702 755 annual live births.^10^

Previous studies of hearing screening for newborns in Thailand have focused on the incidence of and risk factors related to hearing loss,^11^^–^^14^ that is, targeted screening, and not on the effectiveness or potential benefits of a universal screening programme. We therefore implemented a universal newborn hearing screening programme at four sites in southern Thailand to examine the effectiveness and benefit of such a programme. Effectiveness was determined by comparing the outcomes of our study with the benchmarks of the American Academy of Pediatrics:^1^ (i) a screening coverage of at least 95%; (ii) a follow-up of at least 95%; and (iii) a proportion of referrals of at most 4%. We also recorded data on the prevalence of risk factors of hearing loss according to the Joint Committee on Infant Hearing,^4^ and performed a targeted hearing screening analysis. We determined the programme benefit by measuring the composite language scores of infants who were diagnosed with hearing loss because of the screening programme and who received early intervention.

## Methods

### Study design and setting

We conducted our longitudinal descriptive study within the tertiary-care Songklanagarind Hospital, Prince of Songkla University, and the three secondary-care hospitals of Songkhla, Satun and Phatthalung, all located in southern Thailand. Songklanagarind Hospital has a competent audiological service, providing appropriate diagnostic assessment and early intervention. Songkhla, Satun and Phatthalung hospitals are located 21, 105 and 103 km from Songklanagarind Hospital, respectively, and all have effective referral systems. The average birth rate is similar across all four hospitals, at approximately 3000 newborns per hospital per year (unpublished information obtained from obstetricians and gynaecologists in the four hospitals). Before implementing our screening programme, we provided documents describing the background of universal newborn hearing screening and its importance to the administrators, heads of departments and ward staff of these four hospitals.

### Target group

We recruited all infants born during January to July 2017. According to the American Academy of Pediatrics,^1^ the minimal criteria warranting intervention is hearing loss of a moderate degree or greater (> 40 dB hearing loss) at a pure tone average of 0.5–4.0 kHz in the better ear. To maximize our constrained resources, we excluded newborns with a mild degree of hearing loss or with unilateral hearing loss from further screening. The parents of such infants were advised to monitor their child’s hearing and development, and to return for further hearing testing if concerned.

Our target group was newborns with sensorineural hearing loss,^15^ which can be genetic or caused by exposure to certain risk factors. Sensorineural hearing loss is usually permanent and cannot be medically or surgically corrected for; prompt intervention (hearing aid fitting or cochlear implants) is important to avoid developmental delays. However, our screening programme also resulted in the identification of infants with conductive or mixed (a combination of sensorineural and conductive) hearing loss.^15^^,^^16^ Conductive hearing loss can be caused by an ear infection or fluid in the middle ear, for example, and might only require close observation for several months until spontaneous improvement, or grommet insertion. Although we do not include the diagnosed cases of conductive hearing loss (or mixed if the sensorineural hearing loss is only of a mild degree) in prevalence, we consider the benefit of intervention for these cases and include the cost of these interventions in the total cost of the screening programme.

### Technology

For details of the technology used in our programme, please see the data repository.^17^

### Screener recruitment and support

We recruited three nursing assistants (in Songkhla, Phatthalung and Songklanagarind hospitals) and one nurse (in Satun Hospital) to implement our screening programme. We trained our screeners to inform the parents of newborns of the importance of the early detection of hearing loss, provide parents with an information brochure, obtain written consent forms, perform the screening test(s), complete the case record forms, and make appointment dates for further screening and diagnosis, if relevant. Two otologists and a hospital audiologist delivered a 1-day training course at Songklanagarind Hospital for the four screeners. The course covered ear anatomy, basic physiological tests and hands-on training on correct probe insertion in a settled newborn, monitoring stimulus stability, checking response reproducibility and assigning pass/referral outcome. 

The otologists and audiologist were available to support the screeners during the implementation of the programme with instant messaging and video calls via a free smartphone application. The screeners recorded and shared the details of any screening issues and comments, enabling simple and effective communication between the four sites.

### Procedure

Using the case record forms, each screener recorded the demographic data of all newborns as well as their exposure to any risk factors associated with sensorineural hearing loss,^4^ such as a stay in the neonatal intensive care unit, whether the infant had received ototoxic medication or assisted ventilation and whether the infant was being treated for meningitis or an in utero infection. The study participants were recorded as numbers; names (given or family) were not included in the case record forms. The screener evaluated each newborn (older than 1 day) using transient otoacoustic emission technology in a quiet room, either at a well-baby nursery or neonatal intensive care unit. Screeners were also responsible for recording the results of initial screening and, where relevant, of a later second screening, and a diagnostic assessment, any treatment given and composite language scores.

All newborns with any type of moderate to profound bilateral hearing loss were referred for a second screening 1 month later at the ear, nose and throat outpatient clinic of their hospital, conducted by the same screener. Screeners referred infants who failed the hearing test in both ears twice, directly to the audiovestibular clinic of Songklanagarind Hospital for diagnostic assessment and scheduled the appointment with the diagnosing audiologist. Fast-tracking of infants to this diagnostic clinic, without first attending the ear, nose and throat clinic of Songklanagarind Hospital (except in cases such as cerumen impaction or congenital anomalies), was accomplished by the screener writing “UNHS” (universal newborn hearing screening) on an information brochure. The screener instructed parents to take this brochure with them to the diagnostic appointment.

Parents were reminded of their diagnostic appointment a few days in advance by a phone call from the audiologist. Any cancellations were rescheduled and replaced by the next infant in the queue. Parents who failed to attend a scheduled second screening or diagnostic appointment were contacted by telephone and/or text message by their site screener, who recorded their reason for non-attendance.

All infants diagnosed with hearing loss received treatment (e.g. the fitting of a hearing aid for sensorineural hearing loss, or close observation followed by myringotomy with grommet insertion if necessary for conductive hearing loss) and regular follow-up appointments at Songklanagarind Hospital. One of the otologists assessed infants’ speech development at 1 year of age using the Bayley Scales of Infant and Toddler Development, Third Edition, Thai version (Bayley-III, Thai).^18^ This tool was translated and validated from an original English version, and speech and language delays were indicated by a composite language score of below average (< 90).^18^

### Data processing

All data were transferred to an Excel spreadsheet (Microsoft, Redmond, USA) for basic statistical analysis and a review meeting was held to correct any data errors. Two research assistants were recruited for double data entry, and separately entered all data into an Excel spreadsheet. Only the two otologists and the two research assistants could access the password-protected data. For the targeted hearing screening analysis, the filter facility in Excel was used to select only newborns exposed to risks of hearing loss.

### Ethical clearance

Ethical approval was obtained from the Research Ethic Committees of the Faculty of Medicine, Prince of Songkla University before the beginning of the study. Written and informed consent was obtained from the parents of all subjects before they were enrolled in the study.

## Results

**Fig. 1:**
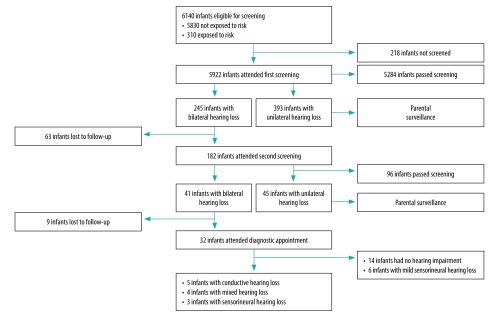
Flowchart of a universal newborn hearing screening programme, Thailand, 2017

Of the total of 6234 live births across the four sites during the study period, 94 neonates died before screening. Of the 6140 infants eligible for screening (Fig. 1), 218 infants missed the screening; 196 were admitted to other wards outside the well-baby nurseries due to patient overload and 22 had severe life-threatening conditions. A total of 245 infants failed the first bilateral hearing screening, but only 182 attended the second screening. The loss to follow-up after the first screening was 25.7% (63/245), yielding an overall coverage of 95.4% (5859/6140).

Of the infants screened a second time, 41 failed the test and were referred to a comprehensive audiological evaluation at the tertiary-care hospital, giving a proportion of referrals of 0.7% (41/6140). Of the infants referred, nine did not attend for diagnostic assessment, resulting in a 22.0% loss to follow-up.

Of the 32 infants attending a diagnostic assessment, the audiologist confirmed hearing loss in 18 infants, comprising five infants with conductive hearing loss, four with mixed hearing loss (sensorineural component of a mild degree) and nine with sensorineural hearing loss (six mild). When only the targeted group is considered, this gives an overall prevalence of less than 0.1% (3/6140). A total of 14 infants failed both screening tests but had normal hearing at the diagnostic assessment, yielding a false-positive proportion of 0.2% (14/6140) for the two-stage screening approach.

In the targeted hearing screening analysis, 310 of the 5922 newborns screened were considered to have been exposed to at least one of the risk factors for hearing loss (Table 1). Of these, one infant (0.3%) was diagnosed with sensorineural hearing loss.

**Table 1:** Infants screened for hearing loss, by risk factor, Thailand, January–July 2017

Risk	No. of infants (%)						
	Exposed to particular risk (*n* = 310)	Bilateral hearing loss detected at first screening (*n* = 245)	Lost to follow-up after first screening (*n* = 63)	Attended second screening (*n* = 182)	Lost to follow-up after second screening (*n* = 9)	Attended diagnostic appointment (*n* = 32)	Diagnosed with sensorineural hearing loss (*n* = 3)
No exposure to risk^a^	NA	187 (76.3)	51 (81.0)	136 (74.7)	0 (0)	25 (78.1)	2 (66.7)
Assisted ventilation	6 (1.9)	0 (0)	0 (0)	0 (0)	0 (0)	0 (0)	0 (0)
Ototoxic medication	75 (24.2)	0 (0)	0 (0)	0 (0)	0 (0)	0 (0)	0 (0)
Anomalies and syndromes associated with hearing loss	6 (1.9)	0 (0)	0 (0)	0 (0)	0 (0)	0 (0)	0 (0)
Stay in neonatal intensive care unit > 5 days	99 (31.9)	0 (0)	0 (0)	0 (0)	0 (0)	0 (0)	0 (0)
**Multiple risks**							
Intensive care, ototoxic drugs and assisted ventilation	94 (30.3)	40 (16.3)	7 (11.1)	33 (18.1)	2 (22.2)	3 (9.4)	0 (0)
Ototoxic drugs and meningitis	15 (4.8)	13 (5.3)	5 (7.9)	8 (4.4)	6 (66.7)	2 (6.3)	1 (33.3)
Ototoxic drugs and in utero infection	15 (4.8)	5 (2.0)	0 (0)	5 (2.7)	1 (11.1)	2 (6.3)	0 (0)

NA: not applicable.

^a^ Number of infants screened: 5612.

Note: The flowchart of the screening process is presented in Fig. 1. Due to rounding, some inconsistencies arise in some values.

Among the 12 infants who received early intervention, nine showed good speech and language outcomes 1 year later (scores of 91–109); the remaining three had poor outcomes (scores of < 90; Table 2).

**Table 2:** Characteristics of those diagnosed with hearing loss because of a pilot hearing screening programme for newborns, Thailand, January–July 2017

Subject	Type of hearing loss	Air conduction threshold in the better ear (dBeHL)	Bone conduction threshold in the better ear (dBeHL)	Exposed to risk of hearing loss	Additional disability	Treatment	Composite language scores at 1 year^a^
1	Conductive	30	Normal	No	No	Observation	103
2	Conductive	40	Normal	Yes	Trisomy 18	Observation	47
3	Conductive	30	Normal	Yes	No	Observation	103
4	Conductive	50	Normal	Yes	No	Observation	109
5	Conductive	55	Normal	Yes	Cleft palate	Myringotomy with grommet insertion	94
6	Mixed	50	35	Yes	Cleft palate	Myringotomy with grommet insertion	91
7	Mixed	≤ 60^b^	35	No	No	Observation	103
8	Mixed	50	35	No	No	Observation	97
9	Mixed	40	30	Yes	Cleft palate	Myringotomy with grommet insertion	94
10	Sensorineural	70	> Maximum levels of 45	Yes	Cerebral palsy	Hearing aid	47
11	Sensorineural	60	> Maximum levels of 45	No	No	Hearing aid	100
12	Sensorineural	90	> Maximum levels of 45	No	No	Hearing aid	47

dBeHL: decibels estimated hearing levels

^a^ We assessed children’s speech and language development by using the Bayley Scales of Infant and Toddler Development, Third Edition, Thai version (Bayley-III, Thai).^18^ A score below 90 indicated a speech and language delay.

^b^ The diagnostic assessment was incomplete as the subject could not sleep calmly during a whole testing session.

The total cost of the screening programme was 26 833 United States dollars (US$) and US$ 4.5 per infant screened (Table 3).

**Table 3:** Costs of a universal newborn hearing screening programme at four hospitals in southern Thailand, January–July 2017

Item	Description and/or assumptions	Total cost (US$)^a^
**Screening**		
Transient evoked otoacoustic emissions machine	Cost of $10 294 per unit; one unit purchased per site	7996^b^
Supplies	Reusable ear tips at US$ 70.50 for one pack of 30 pieces; one pack purchased per site	282
Wages	Screener salary US$ 6 per hour; each screening took an average of 15 minutes per infant (5922 attended first screening)	8883
**Diagnosis**		
Auditory brainstem response and auditory steady-state response machine	Cost of US$ 41 176 per unit; single unit purchased	3998^c^
Diagnostic otoacoustic emissions machine	Cost of US$ 11 765 per unit; single unit purchased	1142^c^
Tympanometry machine	Cost of US$ 7353 per unit; single unit purchased	714^c^
Supplies	Disposable electrodes for testing cost US$ 1.4 per infant; reusable ear tips for otoacoustic emissions and tympanometry testing cost US$ 2.7 per infant (32 infants attended diagnostic appointment)	131
Wages	Audiologist salary US$ 6 per hour; each diagnostic appointment lasted an average of 2 hours per infant (32 infants referred to audiologist)	384
**Intervention**		
Hearing aids	Cost US$ 441 per unit; 3 infants fit bilaterally (6 units)	2646
Myringotomy	Cost US$ 75 per procedure; 3 infants underwent surgery	225
Wages	Speech-language therapist salary US$ 6 per hour; 3 infants had aural rehabilitation, comprising 24 sessions of 1 hour per session	432
**Total expenditure**	NA	26 833
**Cost per infant screened**	NA	4.5^d^
**Cost per infant diagnosed with sensorineural hearing loss**	NA	8944^e^

NA: not applicable; US$: United States dollars

^a^ An exchange rate of 34.00 Thai Baht = US$ 1.00 (1 July 2017) was used to convert costs from local currency.

^b^ Total repayments over 1 year calculated based on the manufacturer’s price per machine, amortized over 5 years at a discount rate of 3%.^19^

^c^ Total repayments over 1 year calculated based on the manufacturer’s price per machine, amortized over 10 years at a discount rate of 3%.^19^

^d^ In total, we screened 5922 infants.

^e^ We diagnosed three infants with sensorineural hearing loss.

## Discussion

In terms of effectiveness, our study only achieved two out of the three American Academy of Pediatrics benchmarks,^1^ that is, coverage and percentage of infants referred. We obtained a screening coverage of 95.4%. Although considered effective by the benchmark, this could have been higher. Almost 200 newborns missed the screening because they were admitted to other wards outside the well-baby nursery. This issue had not been considered when we planned our screening study, as it was believed that all four sites had adequate capacity for all births. This problem could be overcome by assigning a specific person to monitor all births before they are transferred to a well-baby nursery.

We lost almost one quarter of infants to follow-up at different stages of our study, exceeding the benchmark of 5% maximum set by the American Academy of Pediatrics. The main reasons given by parents for failing to attend either a second screening or a diagnostic appointment included: work constraints, a belief that their infants did not have a hearing problem or lack of transport. These reasons indicate that our methods of informing parents of the importance of hearing screening for newborns at the initial stage of the programme were inadequate; this issue could be addressed by providing better maternal education regarding hearing loss during antenatal care. Other methods of increasing the percentage of follow-up include: obtaining details of a designated regular contact (such as a relative or close friend), as well as of parents; using a computer-based system to manage and monitor multiple requests to attend appointments via letter, email and telephone; or scheduling hearing appointments with immunization programmes, which has been shown to improve the follow-up in low- and middle-income countries.^20^ However, the latter strategy should be considered with caution as most newborns in Thailand are scheduled for first immunization (hepatitis B vaccine) at 2 months of age, meaning that the recommendation of the Joint Committee on Infant Hearing^5^ to detect hearing loss and provide intervention before the age of 3–6 months would be difficult to implement. 

Our high loss to follow-up could also have lowered our calculated prevalence of sensorineural hearing loss in newborns (less than 0.1%), which was lower than that reported from Thailand (0.2%, 11/6342),^11^ South Africa (0.1–0.2%)^21^ and other countries (0.1–0.6%).^7^ Our data show that almost one fifth and all of the infants lost to follow-up at the first and second stages, respectively, were exposed to multiple risks of hearing loss; these children were more likely to have a hearing disorder than those who were not exposed to such risks. Any hearing-impaired infants in those lost to follow-up might not have their disability identified until 2 years of age.^22^

Regarding the ≤ 4% referral benchmark of the American Academy of Paediatrics, we obtained a proportion of referrals of 0.7% in our study. We do not believe that our low referral rate was a result of inadequate training of screeners, as it has been shown that 2–4 hours of training in such studies is sufficient.^23^ However, our screening protocol excluded all unilateral referrals. Although not all individuals with unilateral hearing loss require intervention, there is growing agreement to include these newborns in any hearing screening programme.^7^ The proportion of the 393 newborns with unilateral referrals from the first-stage screening who might have failed the second-stage screening and required further diagnostic evaluation is unknown. By expanding our protocol, we may have achieved a slightly higher referral rate.

In terms of benefit, our study demonstrates that prompt intervention provides good speech outcomes for most infants diagnosed at a young age with hearing loss. Despite receiving prompt treatment, three infants had poor speech outcomes. However, two of these infants had the additional disabilities of trisomy 18 with global delayed development and generalized cerebral palsy with cognitive impairment, which are barriers to the development of normal speech regardless of hearing ability.^24^^,^^25^ The third infant had profound deafness that even powerful hearing aids could not improve; in such cases, cochlear implantation is recommended.^26^

Researchers have shown in Egypt^27^ and India^28^ that targeted newborn hearing screening is another valuable option, especially when resources are constrained.^7^ However, our targeted analysis yielded a very low prevalence (0.3%) compared with other targeted screening studies (2.0–4.0%).^1^^,^^2^ As well as high loss to follow-up, our low prevalence may have been a result of our selected hearing screening technology. We used transient evoked otoacoustic emission for our screening technology, due to its ease of use and reasonable cost, but this method can only detect hearing loss caused by cochlear lesions. Auditory neuropathy spectrum disorder, a type of sensorineural hearing loss that is typically found in infants who require intensive care,^29^ is caused by retrocochlear lesions.^30^ Although automated auditory brainstem response technology can detect both cochlear and retrochochlear lesions, this technology is more expensive and requires longer screening appointments. We can report that, of the 5284 infants who passed the first hearing screening and were not assessed further, 88 had been looked after in the neonatal intensive care unit for a period of longer than 5 days. We cannot know how many of these 88 newborns may have been diagnosed with a hearing disorder if we had used the more expensive technology.

As well as informing policy-makers, our study benefited from good communication between the four hospitals involved, an efficient fast-tracking service for participants and the low cost calculated per infant screened for the programme.^19^ The low cost is similar to the routine screening of newborns for phenylketonuria or hypothyroidism in Thailand. The prevalence of sensorineural hearing loss is similar to that of hypothyroidism (less than 0.06%; 1/1690) and much greater than that of phenylketonuria (0.0004%; 1/223 735).^31^ Another reason for encouraging universal screening, despite the lower cost of targeted hearing screening, is that about half of all children diagnosed with a hearing impairment are actually exposed to the risks of hearing loss.^7^ We also argue that a cost–effectiveness analysis should be conducted and that the cost of universal newborn hearing screening should be estimated in terms of cost per quality-adjusted life-year.

Although we did not demonstrate the complete effectiveness of universal screening, the confirmed benefits of early intervention, in terms of speech and language development, warrant further research and increased efforts to procure the required public health funding.
